# TIMELESS upregulates PD-L1 expression and exerts an immunosuppressive role in breast cancer

**DOI:** 10.1186/s12967-023-04257-6

**Published:** 2023-06-20

**Authors:** Xinrui Dong, Huijuan Dai, Yanping Lin, Xiaonan Sheng, Ye Li, Yaohui Wang, Xueli Zhang, Shuheng Jiang, Wenjin Yin, Jinsong Lu

**Affiliations:** 1 Department of Breast Surgery, Renji Hospital, School of Medicine, Shanghai Jiaotong University, No. 1630 Dongfang Road, Shanghai, 200127 China; 2 State Key Laboratory of Oncogenes and Related Genes, Shanghai Cancer Institute, Renji Hospital, School of Medicine, Shanghai Jiaotong University, Shanghai, 200240 China

**Keywords:** TIMELESS, PD-L1, CD8^+^ T lymphocytes, Breast cancer

## Abstract

**Background:**

Upregulation of the PD-L1 (CD274) immune checkpoint ligand on the tumor surface facilitates tumor immune escape and limits the application of immunotherapy in various cancers, including breast cancer. However, the mechanisms underlying high PD-L1 levels in cancers are still poorly understood.

**Methods:**

Bioinformatics analyses and in vivo and in vitro experiments were carried out to assess the association between CD8^+^ T lymphocytes and TIMELESS (TIM) expression, and to discover the mechanisms of TIM, the transcription factor c-Myc, and PD-L1 in breast cancer cell lines.

**Results:**

The circadian gene TIM enhanced PD-L1 transcription and facilitated the aggressiveness and progression of breast cancer through the intrinsic and extrinsic roles of PD-L1 overexpression. Bioinformatic analyses of our RNA sequencing data in TIM-knockdown breast cancer cells and public transcriptomic datasets showed that TIM might play an immunosuppressive role in breast cancer. We found that TIM expression was inversely associated with CD8^+^ T lymphocyte infiltration in human breast cancer samples and subcutaneous tumor tissues. In vivo and in vitro experiments demonstrated that TIM knockdown increased CD8^+^ T lymphocyte antitumor activity. Furthermore, our results showed that TIM interacts with c-Myc to enhance the transcriptional capability of PD-L1 and facilitates the aggressiveness and progression of breast cancer through the intrinsic and extrinsic roles of PD-L1 overexpression. Moreover, public database analysis suggested that high TIM levels were positively related to PD-L1 inhibitor therapeutic response.

**Conclusions:**

Mechanistically, we first found that TIM could upregulate PD-L1 by interacting with c-Myc to enhance the transcriptional capability of c-Myc to PD-L1. Altogether, our findings not only provide a novel therapeutic strategy to treat breast cancer by targeting the oncogenic effect of TIM but also indicate that TIM is a promising biomarker for predicting the benefit of anti-PD-L1 immunotherapy.

**Supplementary Information:**

The online version contains supplementary material available at 10.1186/s12967-023-04257-6.

## Background

Breast cancer is the most common cancer among women worldwide [1]. Although the breast cancer death rate has been decreasing largely owing to substantial advances in therapeutic approaches, a considerable number of patients with metastases remain resistant to existing treatments. These alarming data make it urgent to explore innovative therapeutic avenues for breast cancer patients [2]. In recent years, immunotherapy has revolutionized the management of many malignant tumors, including breast cancer [2], although it has historically been considered a low immune-reactive tumor compared with other solid tumors [3]. Due to the development of immune checkpoint inhibitors (ICI), immunotherapy for breast cancer has progressed tremendously over the past few years [4, 5]. PD-L1, a well-known inhibitory molecule, interacts with PD-1 on CD8^+^ T lymphocytes, enabling cancer cells to escape immune surveillance and shut down antitumor immunity [6]. Tumors with high PD-L1 expression generally exhibit a better response to ICI treatment [3]. Therefore, there is a need to identify immunosuppressive factors capable of elevating PD-L1 expression to improve ICI response.

Malignant tumors evade immune attacks by upregulating PD-L1 expression in cancer cells [7]. Elevated PD-L1 expression has been proven to influence cancer cells from intrinsic tumor function to extrinsic immune environment changes, encompassing the stimulation of tumor cell proliferation and survival and the suppression of cytotoxic T lymphocyte (CTL) function [8, 9]. Recent studies have shown that PD-L1 expression is regulated by diverse mechanisms. Deubiquitination of PD-L1 by OTUB1 promotes the degradation of PD-L1 through the endoplasmic reticulum-associated degradation (ERAD) pathway, facilitating CD8^+^ T lymphocyte infiltration and increasing cytotoxicity ability [9]. Palmitoylation of PD-L1 by palmitoyltransferase ZDHHC3 restrains PD-L1 ubiquitination and degradation, consequently suppressing tumor-specific T-cell immunity [10]. Moreover, glycosylation of PD-L1 by glycogen synthase kinase 3b (GSK3b) reduces anti-tumor T-cell activity [11]. Besides, the abundance of PD-L1 was also mediated by the amplification of many transcription factors targeting the encoding CD274 gene locus, such as c-Myc [12], STAT [13] and NF-κB [14]. However, a comprehensive picture of PD-L1 regulators remains far from fully understood.

Circadian rhythms are associated with various biological and physiological processes in mammals that maintain homeostasis [15]. Disruption of the circadian rhythm plays a crucial role in the pathogenesis of cancers, including breast cancer [16]. Circadian rhythms can be disrupted by various factors, including alterations in clock genes. Therefore, targeted modulation of core clock genes may be a new approach to cancer treatment [17]. TIMELESS (TIM) is a core clock gene [18] that contributes to carcinogenesis in many cancers. Some studies have demonstrated that TIM can promote the progression of breast cancer by dysregulating the self-renewal of stem cells and the stability of replication forks [19–21]. Our previous research has confirmed that TIM can promote the growth of breast cancer through sphingolipid synthesis [22]. It also affects the immune status of ovarian cancer cells by reducing macrophage infiltration [23]. However, the role of TIM in antitumor immunity against breast cancer and the underlying mechanism are still unknown and warrant further exploration.

Studies have shown that circadian rhythm disorders can reshape tumor microenvironment (TME) cells and their components by coordinating gene expression, reprogramming cell metabolism, and abnormally activating signaling pathways [24, 25]. In this study, we demonstrated that TIM could affect the immune response in breast cancer by analyzing our transcriptome sequencing data and the breast cancer TCGA database. We further found that increased TIM expression significantly correlated with decreased infiltration of CD8^+^ T cells in breast cancer tissues. In vivo and in vitro experiments have demonstrated that TIM suppresses CD8^+^ T cell immune activity. Moreover, we uncovered the mechanism through which TIM regulates PD-L1 expression by interacting with c-Myc. Collectively, our work sheds a refreshing insight into breast cancer immunotherapy, and TIM might be a potential biomarker for immunotherapy in patients with breast cancer.

## Methods

### Data availability

The cancer genome atlas (TCGA) datasets were obtained by the R package “TCGAbiolinks” and divided into two parts using the median expression of TIM as a cutoff. One thousand ninety-seven samples of breast cancer were collected. Counting data is converted to transcripts per million (TPM) and normalized log_2_ (TPM + 1) while keeping clinical information intact. The data on survival time and clinical characteristics were also obtained. The RNA sequencing data was the same in the article as our lab previously published. The NCBI (National Center for Biotechnology Information)-GEO datasets GSE161529, and GSE130472 and their figures were obtained from TISCH and TISMO datasets. Data of GSE46141 was obtained from NCBI.

### Cell culture

Both human and mouse breast cancer cell lines were obtained from Renji Hospital, Shanghai Jiaotong University, School of Medicine, including MCF-7, T-47D, MDA-MB-231, BT-20, and 4T1. All the cells except MDA-MB-231 and T-47D were maintained in DMEM medium (Gibco, USA), supplemented with 10% Fetal Bovine Serum (Gibco, USA) and 1% Penicillin–Streptomycin (Gibco, USA). MDA-MB-231 was maintained in Leibovitz's 15 medium (Gibco, USA) while T-47D was maintained in RPMI 1640 (Gibco, USA), both supplemented with 10% Fetal Bovine Serum (Gibco, USA) and 1% Penicillin–Streptomycin (Gibco, USA). Cells were cultured at 37 ℃ with 5% CO_2_.

### Antibodies

Western blotting: TIMELESS (Abcam, ab109512, 1/50000), β-actin (Abcam, ab49900, HRP-linked,1/50000), c-Myc (Abcam, ab32072, 1/1000) and PD-L1 (Cell Signaling Technology, #13684, 1/1000), Rabbit secondary antibody (Cell Signaling Technology, #7074, 1/2000).

Immunohistochemistry: TIMELESS (Abcam, ab72458, 1/200), CD8a (Servicebio, GB13429, 1/200), PD-L1 (Servicebio, GB11339A, 1/500), Ki-67 (Servicebio, GB111141, 1/500), Mouse secondary antibody (Servicebio, GB23301, 1/200), Rabbit secondary antibody (Servicebio, GB23303, 1/200).

Immunofluorescence: TIMELESS (Abcam, ab109512, 1/100), c-Myc (Abcam, ab32072, 1/100), CD8a (Servicebio, GB13429, 1/5000), Granzyme B (Abcam, ab255598, 1/2000), active caspase 3 (Servicebio, GB11532, 1/500), Hoechst 33342 (Beyotime, C1027, 1/100), CY3-TSA (Apex Bio, K1051, 1/200), Donkey Anti-Rabbit IgG H&L Alexa Fluor^®^ 647 (Abcam, ab150075, 1/200).

Flow cytometry: PD-L1 (Cell Signaling Technology, #13684, 1/200), CD8a-PerCP/Cyanine5.5 (Clone 53-6.7, Biolegend 1007341/100), CD45-AF700 (Biolegend 103128, 1/80), Fixable Viability Dye eFluor™ 450 (Invitrogen, 1/500).

### Cell transfection and infection

To knock down TIM, the small interference RNAs (siRNAs) siTIM, sic-Myc and its control of both humans and mice were purchased from Genepharma Shanghai, China, and transfected using Polyplus Transfection jetPRIME (France) according to the manufacturer’s instructions. After 48 h, cells were collected for the following research. To stably knock down TIM (shTIM), cells were infected with lentivirus vectors with short hairpin RNAs (shRNA) targeting mouse TIM sequences and empty plasmid as control. To stably overexpress TIM (oeTIM), the human TIM cDNA was cloned into the lentiviral expression vector GV341 to construct the vector GV341-hTIMELESS. The recombinant lentivirus was purchased from Genechem Shanghai, China, and used according to the protocol. After infection, puromycin (0.5 µg/ml, Solarbio) was used to select stably transduced cells. All the RNAs and plasmid sequences are listed in Additional file 1: Tables S1 and S2.

### Human samples and immunohistochemistry (IHC) staining

A cohort of 30 formalin-fixed paraffin-embedded human breast cancer specimens was used. Breast cancer tissues were collected at Renji Hospital during 2021–2022. IHC staining was performed using 5 μm sections of the formalin-fixed paraffin-embedded human breast cancer cutting onto glass slides. IHC for each protein was performed with the anti-TIM and anti-Ki-67 antibody on tumor cells, anti-CD8a antibody on lymphocytes, and PD-L1 on tumor and lymphocytes according to the standard protocol. The relative expression levels of TIM and Ki-67 were calculated by the percentage of the positive cells multiplied by the intensity of protein expression and finally normalized as percentage. The ratio of the positive cells was estimated for CD8a. Five areas for each slide were calculated into quantitative analysis.

### In vivo syngeneic transplanted breast tumor models

Female BALB/c mice (aged five weeks) were obtained from Shanghai Charles River Animal Co., Ltd. Mice were grouped into four animals. They were maintained according to the criteria outlined in the Guide for the Care and Use of Laboratory Animals (23 ℃, 50% humidity, 12/12 h light/dark). 1 × 10^6^ treated 4T1 cells in 100 μL PBS were subcutaneously injected into the fat pad of the 4th mammary gland of the mice. The tumor diameters were monitored every five days. Tumor volumes (V) were measured using the long diameter (a) and the perpendicular diameter (b): V = a × b^2^/2. Mice were sacrificed at the end of the experiment or once the tumor tissue volume reached 1500 mm^3^. Then the tumor was isolated, weighed, and treated for the following experiments.

### RNA extraction and quantitative real-time PCR (RT-qPCR)

The total RNA of cells or tissues was extracted by SimplyP Total RNA Extraction Kit (BioFlux, USA). Total RNA was resuspended in RNase-free water in the kit. The RNA concentration was determined by spectrophotometry using a Nanodrop device (Thermo scientific Nanodrop 2000/2000c, wavelength between 230 to 340 nm). RNA expression levels were quantified by RT-qPCR. Reverse transcription was performed using 200 ng RNA with the HiScript II Q Select RT superMix for the qPCR kit (Vazyme, R233-01). RT-qPCR was performed on 2 ng of cDNA samples using Light Cycler 480 SYBR Green I Master Mix (Roche) supplemented with 0.625 μM forward primer and 0.625 μM reverse primer, and fluorescence was measured by Light Cycler 480 II instrument (Roche). For RNA expression levels, relative quantification was performed using the 2^−ΔΔCt^ method. β-actin was used for internal reference. Sequences of the primers can be found in Additional file 1: Table S3.

### Protein extraction and Western blot

Cell samples were lysed in fresh RIPA Lysis Buffer 10X (Beyotime, P0013C) with protease inhibitor cocktail 100X (Sigma) and phosphatase inhibitor cocktail 100X (Sigma). Western blot membranes were incubated with the corresponding primary antibodies. β-actin was used as a loading control. Rabbit secondary antibody was used. Enhanced chemiluminescence (ECL) was performed by Immobilon Western Chemiluminescence HRP Substrate (Millipore, Billerica, USA) with ChemiDoc Touching Imaging System (Bio-rad, California, USA) and finally quantified by Image Lab software (Version 6.0.1, Bio-rad). During quantification, β-actin was used for internal reference.

### Cell counting kit-8 (CCK-8) assay and clonal formation assay

CCK-8 and clonal formation assays were performed as previously described [22].

### Transwell migration and transwell invasion assay

In the transwell migration assay, 2 × 10^4^ cells were suspended in 100 µl serum-free DMEM in the top compartment of the transwell chamber (Corning). In the lower compartment, 800 µl of DMEM with 10% Fetal Bovine Serum was added, and siRNA was added 24 h before adding cells to the chambers. Once the cells had been fixed with 4% paraformaldehyde for a further 24 h, they were stained with crystal violet, and the non-migrating cells were carefully removed. Images were taken using a light microscope at 20 × magnification. Transwell invasion assay was performed the same way as transwell migration assay, except matrigel was added to the upper chamber.

### Tumor infiltration cell isolation and flow cytometry analysis

Cells were digested and regenerated into single-cell suspensions. Then cells were incubated with the corresponding first antibodies for 30 min and second antibodies for 30 min in the dark and detected by flow cytometry. 0.1% Triton X-100 (Sigma-Aldrich) was used for intranuclear staining. When detecting the immune cells in tumor tissue, after being separated from the mice, the tumor was minced with scissors and dissociated with collagenase IV (Sigma) at 1 mg/ml and collected by a 70 μm filter. After the digestion mixture was centrifuged and regenerated into suspensions by PBS, ficoll (GE healthcare, Ficoll-Paque PLUS) was added to the suspensions and centrifuged. Only the cells in the medium layer were picked for the following experiment. Cells were washed and data were acquired by Fortessa X-20 flow cytometry (BD Biosciences). All the flow cytometry data were processed with FlowJo software (version 10.4).

### Immunofluorescence

Cells, frozen sections, or paraffin sections were fixed with 4% paraformaldehyde (PFA) and washed with PBS 3 times. For tumor mass, 5 μm sections of the mass were cut. Cells or slides were stained with Hoechst stain and corresponding antibodies as shown in the Antibodies subsection in Materials and Methods section according to the manufacturers’ protocols. Fluorescent images were captured using a Leica DM2500 microscope.

### In vivo tumor cell line specific CD8^+^ cytotoxic T lymphocyte (CTL) generation and co-culture

BALB/c female mice aged 8 weeks were sacrificed and their spleens were used to separate CD8^+^ T cells by mouse CD8a (Ly-2) microbeads isolation kit (Miltenyi Biotec, USA) according to the manufacturer's protocol. For tumor-specific CD8^+^ CTL generation, the purified CD8^+^ T cells were cultured in 5 μg/ml CD3 (Biolegend, USA) and 5 μg/ml CD28 (Biolegend, USA) in RPMI 1640 with 10% heat-inactivated FBS at 37 ℃ in 5% CO_2_ for 72 h to generate CTLs. Then the CD8^+^ T cells and CTLs were mixed with tumor cells at a ratio of 10:1 and were cultured in RPMI 1640 with 10% heat-inactivated FBS at 37 ℃ in 5% CO_2_ for 48 h for the following experiments. Annexin V-AbFluor™-488 /PI apoptosis detection kit was purchased from Abbkine (USA) and detected according to the protocol.

### Co-immunoprecipitation (co-IP) assay

The immunoprecipitation was done by a classic magnetic protein A/G IP/Co-IP Kit (YJ201, epizyme). Cells on 10 cm culture dishes were washed three times with PBS and lysed in 0.7 ml lysis buffer, and protease inhibitor mixture (Sigma). Insoluble material was removed after centrifugation at 4 ℃ for 15 min, 12000 rpm. Antibody for immunoprecipitation of each protein was added to the cell lysates and incubated for 1 h at room temperature. Then the antibody-antigen complex was incubated with protein‐A/G‐agarose beads for one hour at room temperature. Then the immunoprecipitated proteins were washed with lysis buffer and then denatured with loading buffer at 100 ℃ for 10 min. Then samples were used to make a Western blotting analysis as mentioned before.

### Cell suspension cytokine detection analysis

The suspension of cells was collected, and the IFN-γ was assessed by the IFN-γ mouse uncoated ELISA kit (EK280/3-96, liankebio) according to the product instructions.

### Detection of cell apoptosis level by double staining flow cytometry

Cells were digested with trypsin without EDTA and resuspended into 1 × 10^6^ cells per group. Then cells were treated with the Annexin V-AbFluor™ 488/PI Apoptosis Detection Kit (KTA0002, Abbkine) according to the product instructions.

### Gene set enrichment analysis (GSEA)

GSEA analysis was carried out with the software GSEA (version 4.2.3) and with the R package “clusterProfiler”. The Hallmark gene sets, Gene Ontology gene sets, REACTOME gene sets, BIOCARTA gene sets, and KEGG (Kyoto Encyclopedia of Genes and Genomes) gene sets were used as the molecular signatures databases “MSigDB Collections”. The core enrichment gene expression plot was also obtained by GSEA software. The circle plot of GSEA was drawn by R package “clusterProfiler”, “ggplot2”, “circlize”, “grid”, “graphics” and “ComplexHeatmap”. The R software version was R 3.6.3.

### MCPcounter cell types enrichment analysis and single sample gene set enrichment analysis (ssGESA)

Estimation of immune fractions of TIM was determined through breast cancer tissue in the TCGA database using MCPcounter by R package “ebecht/MCPounter”. The ssGESA was also applied to explore the different infiltration degrees of the immune cells by R package “GSVA”. The data was tested by the Student’s t-test and visualized using R software.

### Tumor immune dysfunction and exclusion (TIDE) analysis

Potential ICI response was predicted with the TIDE algorithm (http://tide.dfci.harvard.edu/). The χ^2^ test was calculated and visualized using R software.

## Results

### TIM is a potential immunosuppressive target in breast cancer and may be negatively associated with CD8^+^ T lymphocyte infiltration in breast cancer

**Fig. 1:**
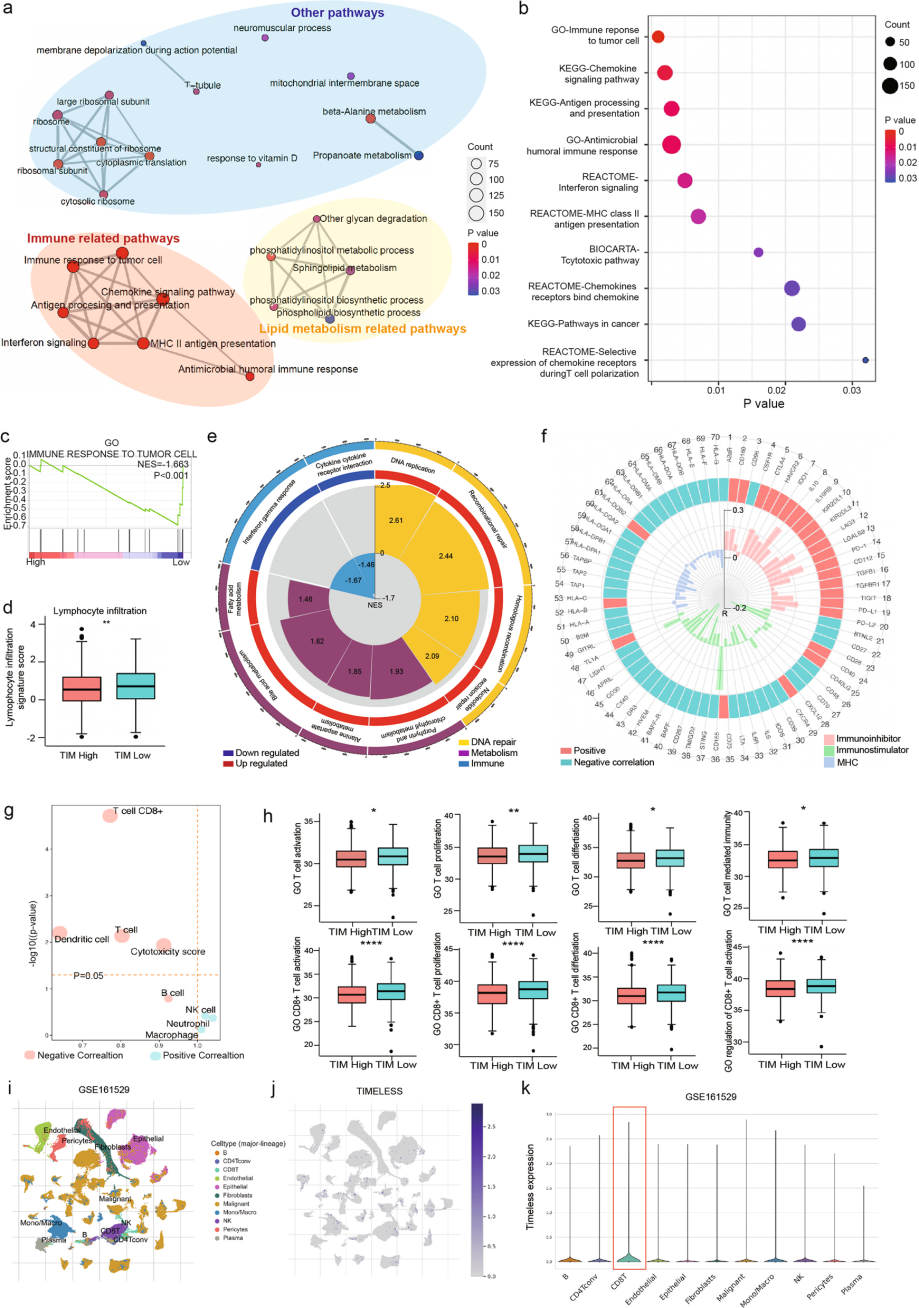
Both our RNA-seq data and the bioinformation datasets analyses showed that TIM is associated with immunogenicity, especially with CD8^+^ T cells. **a** GSEA analysis of differential gene profiles between TIM siRNA transfected breast cancer MCF-7 cells and controls. There are immune-related pathways, lipid metabolism-related pathways, and other pathways. **b** Top 10 immune-related pathways in GSEA analysis with our RNA-seq data. **c** The detailed figure of top GSEA enrichments related to GO immune response to the tumor cell in MCF-7 siTIM group. **d** Comparison of lymphocyte infiltration signature score between TIM high and low expression groups with TCGA BRCA dataset. **e** GSEA analysis of differential gene profiles between the TIM high and low expression groups in the TCGA BRCA dataset. The GSEA enrichment circle plot showed the category of pathways, detailed pathway names, regulation tendency, and enrichment score. Three major types of pathways were enriched: DNA repair-, metabolism-, and immune-related pathways. **f** Immunophenogram for the visualization of terms determining antigen processing (MHC), immune stimulation (immunostimulators), and immune suppression (immunoinhibitors). **g** Volcano plot for the immune subsets in BRCA of TCGA dataset based on the MCPcounter immune score. **h** Boxplots of different T cell- or CD8^+^ T cell-associated functions based on the ssGESA immune algorithm of the TCGA dataset. **i** UMAP visualization of the total cells profiled in GSE161529, with each cell color-coded for the associated cell type. **j**TIM expression in each cell type in the UMAP visualization of GSE161529. **k** Villon plot of TIM expression in each cluster of cells of GSE161529 within the UMAP algorithm. Significance was tested as described in “Methods” section: *p-value < 0.05; **p-value < 0.01; ***p-value < 0.001; ****p-value < 0.0001. *TIM* TIMELESS, *GSEA* Gene Set Enrichment Analysis, *BRCA* breast cancer, *MHC* major histocompatibility complex, *UMAP* Uniform Manifold Approximation and Projection

Our previous study revealed that TIM interacts with specificity protein 1 (Sp1) and upregulates sphingolipid synthesis, which contributes to breast cancer growth and activates mitochondrial respiration [22]. To further elucidate the potential mechanisms of TIM in breast cancer progression, we performed GSEA using RNA-seq data from TIM-knockdown MCF-7 cells and the corresponding control cells. GSEA indicated that TIM may be involved in orchestrating lipid metabolism-related pathways, immune-related pathways, and several other pathways (Fig. 1a). Further in-depth GSEA analysis based on the immune pathways showed that TIM downregulation was related to the immune responses to the tumor cell, chemokine signaling pathway, antigen processing, and presentation (Fig. 1b). The expression of core immune-related genes was enriched in the siTIM group (Additional file 1: Fig. S1a). The expression profiles of the core enrichment genes in each top-scoring pathway are shown (Fig. 1c; Additional file 1: Fig. S1b). We also performed GSEA using the TIM profile of the TCGA breast cancer database. The lymphocyte infiltration signature score was significantly lower in the TIM high group (Fig. 1d). Taken together, these results demonstrate that TIM plays a vital role in immunosuppression, from antigen presentation to immune attack. GSEA also revealed that DNA repair and metabolism-related signaling pathways were significantly enriched (Fig. 1e). In addition to these two functions, which have already been discovered in existing research, immune-related pathways were also enriched in accordance with our RNA-sequencing data.

Moreover, interferon-gamma response and cytokine-cytokine receptor interactions were downregulated in the high TIM expression group (Additional file 1: Fig. S1c). GSEA showed that TIM suppresses tumor-extrinsic factors such as chemokines that regulate T cell recruitment. To deepen our understanding of the intrinsic immune profiles of TIM, an immunophenogram was constructed based on three categories with their representative genes or gene sets: major histocompatibility complex (MHC) molecules, immunostimulators, and immunoinhibitors (Fig. 1f). The immunophenogram showed that TIM expression was positively correlated with immunoinhibitors and inversely associated with immunostimulators and MHC molecules. Other studies have also revealed the suppressive role of TIM in the regulation of immune functions. For example, other intrinsic immune factors such as tumor mutant burden (TMB) and neoantigen load (NAL) also showed higher expression levels in the high TIM group (Additional file 1: Fig. S1d).

To further reveal the potential immunosuppressive mechanisms of TIM, we examined the immune infiltration landscape of multiple immune cell types in high- and low-TIM groups. Subsequently, we evaluated the immune correlation using the MCPcounter algorithm, which revealed that CD8^+^ T lymphocytes were most significantly and inversely correlated with TIM expression (Fig. 1g). The infiltration levels of T cells and myeloid dendritic cells and cytotoxicity scores also decreased considerably in the high TIM group (Additional file 1: Fig. S1e). TIM expression had a stronger inverse correlation with CD8^+^ T lymphocytes (r = − 0.160, p = 0.021) than with T lymphocytes (r = − 0.061, p = 0.043) in the scatter plot (Additional file 1: Fig. S1f). The ssGESA algorithm was used to confirm the role of TIM in the breast cancer immune microenvironment. Based on ssGESA, we found that T cell activation, proliferation, differentiation, and T cell-mediated immunity were lower in the high TIM group. This result was consistent with that of CD8^+^ T lymphocytes, with a more significant p-value (Fig. 1h). Furthermore, using the TISCH database, we found that TIM was expressed at the highest level in CD8^+^ T lymphocytes among all cell clusters in the single-cell sequence data of GSE161529 (Fig. 1i–k). Taken together, we hypothesized that oncogene TIM promotes breast cancer progression by inhibiting CD8^+^ T lymphocyte infiltration.

### TIM inhibits CD8^+^ T lymphocyte infiltration in human breast cancer tumor tissues

**Fig. 2:**
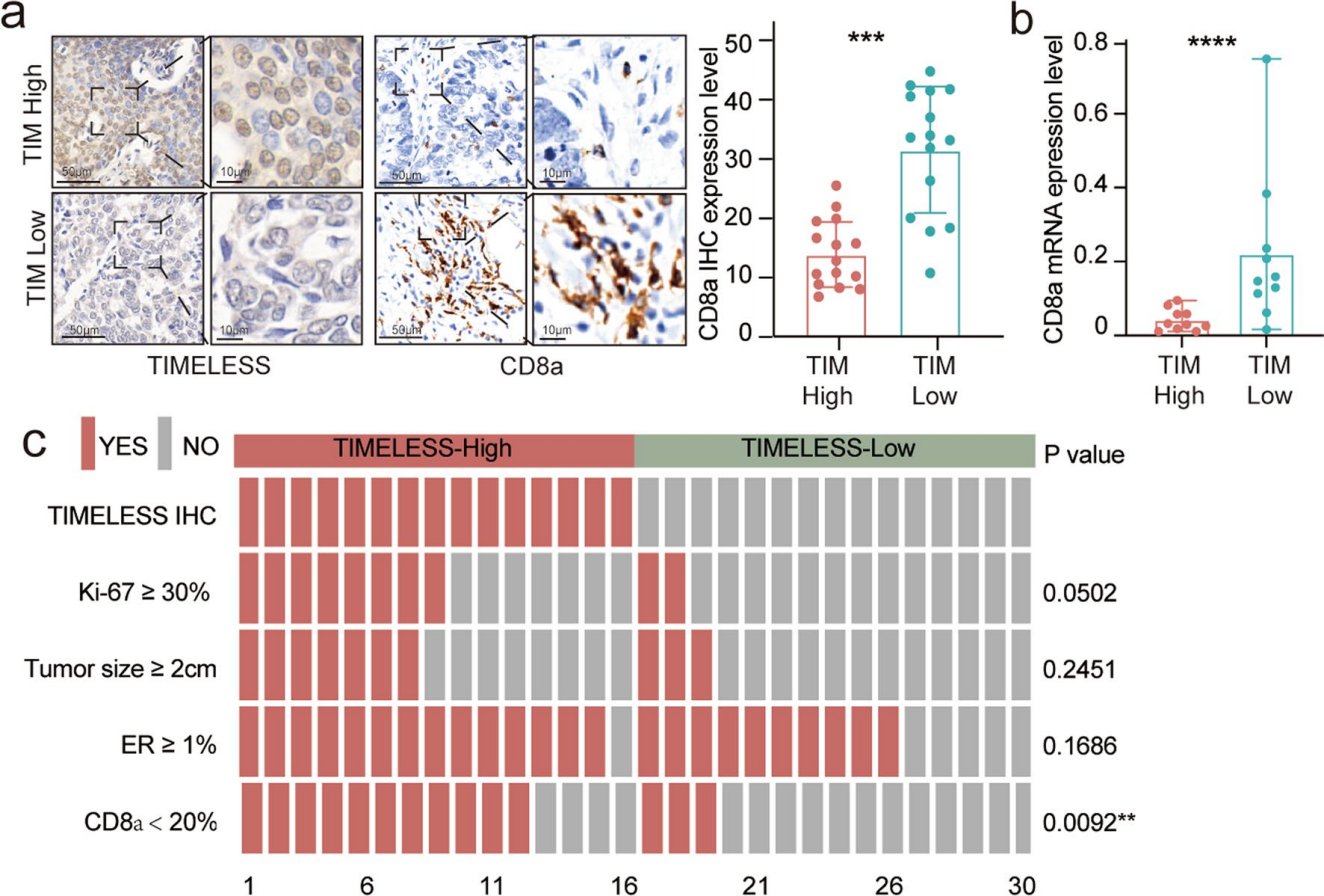
TIM expression inhibits CD8^+^ T lymphocyte infiltration in the RENJI breast cancer patient cohort. **a** Representative CD8a immunohistochemical (IHC) images based on different TIM expression levels. The scale bar is 50 μm. **b** Relative mRNA expression level of CD8a and TIM by RT-qPCR. β-actin was used as the internal reference. Significance was tested by Student’s t-test. **c** Heatmap plot of the clinicopathological features in patients with different TIM expression levels. Statistical significance was performed by the χ^2^ test. Significance was tested as described above: **p-value < 0.01; ***p-value < 0.001; ****p-value < 0.0001. *IHC* Immunohistochemistry, *RT-qPCR* quantitative real-time PCR, *ER* estrogen receptor

We then validated TIM expression using CD8^+^ T lymphocyte infiltration levels in the RENJI breast cancer patient cohort. Both protein levels determined by IHC (Fig. 2a) and mRNA levels determined by RT-qPCR (Fig. 2b) of tumor tissues showed that higher TIM expression was associated with lower CD8a expression. We also evaluated and compared the pathological and clinical features of the RENJI cohort. We found that TIM expression inversely correlated with CD8^+^ T lymphocyte infiltration (Fig. 2c), which is in line with our results (Fig. 1g–k). TIM expression was positively correlated with Ki-67 expression (Fig. 2c).

### TIM inhibits CD8^+^ T lymphocyte infiltration and cytotoxic antitumor activity in vivo and in vitro

**Fig. 3:**
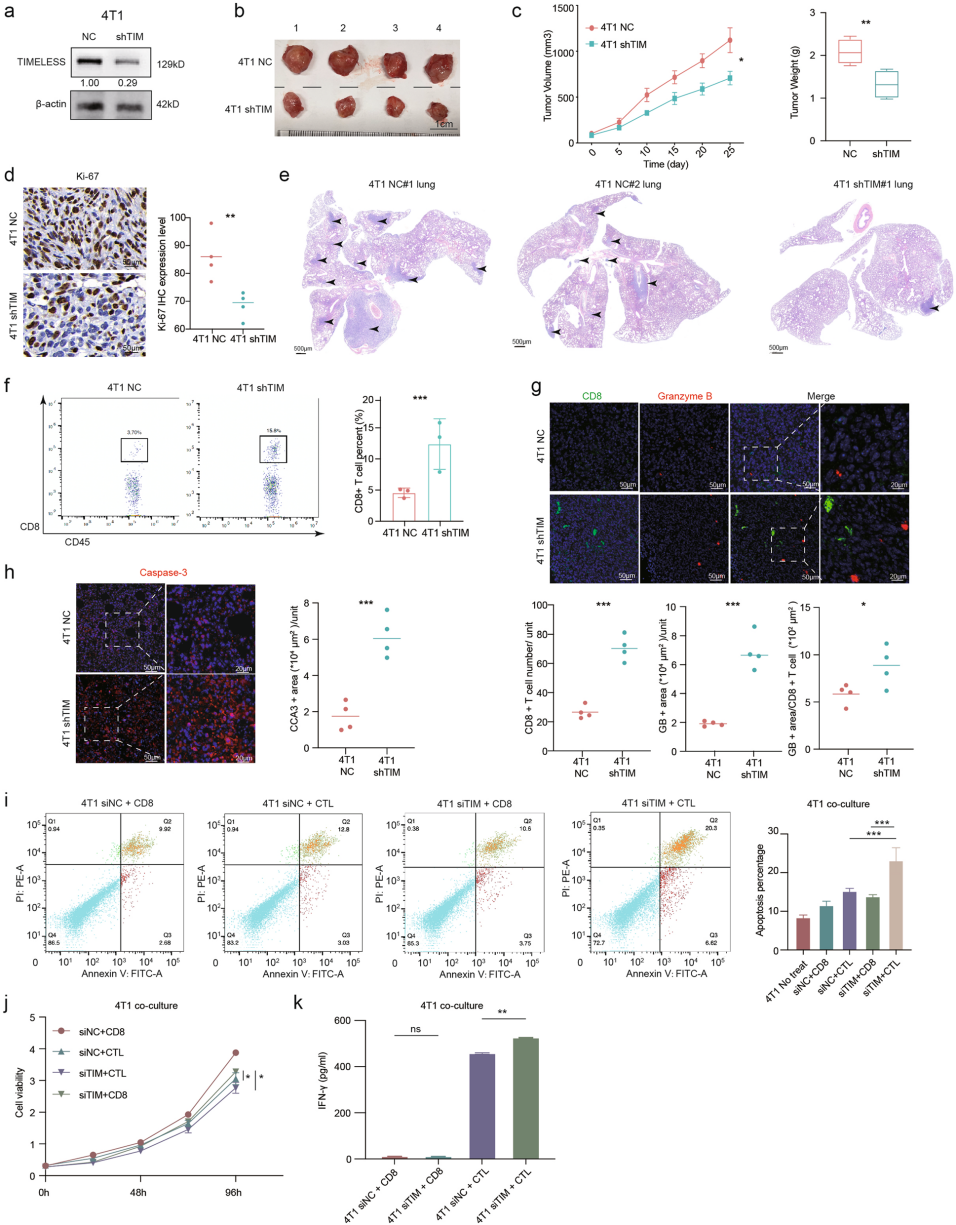
TIM knockdown inhibits breast cancer oncogenic behavior through enhancing CD8^+^ cytotoxic T lymphocytes (CTLs) infiltration in vivo and in vitro. **a** The Western Blot analyses showed the stable interference efficiency of TIM via shRNA in mouse TNBC cell line 4T1. **b** Image of syngeneic tumors at the end of the experiment (4T1 shNC and shTIM groups, n = 4 in each group). The scale bar is 1 cm. **c** Tumor volume in each group was measured every five days and tumor weight at the end of the experiment was measured in each group. Values are means ± SD. **d** Representative Ki-67 IHC images of the syngeneic mice tumor tissue in 4T1 shNC mice and 4T1 shTIM mice. The scale bar is 50 μm. **e** The hematoxylin–eosin staining of lung metastasis in the 4T1 NC group and the 4T1 shTIM group of syngeneic mice. The scale bar is 100 and 500 μm. **f** Flow cytometry analysis was used to evaluate the percentage of CD8^+^ T cells in syngeneic mice tumor tissue from 4T1 shNC mice and 4T1 shTIM mice (n = 3). **g** Immunostaining of CD8 (CTL marker, pseudo-color: green) and granzyme B (activity of T cell, pseudo-color: red) in the 4T1 NC mice and 4T1 shTIM mice. Hoechst: nuclear counterstaining (pseudo-color: blue). The scale bar is 20 and 50 μm. **h** Immunostaining of cleaved caspase 3 (CCA3, pseudo-color: red) in the 4T1 NC mice and 4T1 shTIM mice. Hoechst: nuclear counterstaining (pseudo-color: blue). The scale bar is 20 and 50 μm. **i** Representative flow cytometric analysis figures of apoptosis levels on mouse breast cancer cell line 4T1 co-cultured with their specific CTLs or CD8^+^ T cells in the siNC group and siTIM group. Significant differences in the average apoptosis percentage were seen between siTIM + CTL group and siTIM + CD8 or siNC + CTL group. **j** Cell viability curve of 4T1 co-cultured with their specific CTLs or CD8^+^ T cells in the siNC group and siTIM group. Significant differences were seen between siTIM + CD8 group and siTIM + CTL or siNC + CTL group. **k** Enzyme-linked immunosorbent assay (ELISA) for IFN-γ secretion by CD8 + CTLs after co-culture of 4T1 with their specific CTLs or CD8^+^ T cells in the siNC group and siTIM group. Significance was tested using Mann–Whitney U-test: ns, p-value no significance; *p-value < 0.05; **p-value < 0.01; ***p-value < 0.001. *TNBC* triple negative breast cancer, *IHC* Immunohistochemistry, *CTL* cytotoxic T lymphocytes, *CCA3* cleaved caspase 3, *ELISA* enzyme-linked immunosorbent assay

To investigate the effect of TIM on tumor immunity, we established a subcutaneous tumor model of breast cancer using the mouse breast cancer cell line 4T1 in female BALB/c mice with immune integrity. TIM expression was stably knocked down in mouse shTIM 4T1 cells (Fig. 3a). We subcutaneously injected shTIM and shNC 4T1 cells in female BALB/c mice aged five weeks. Tumor growth was significantly suppressed in shTIM syngeneic mice compared to that in the shNC group (Fig. 3b–c). IHC staining also suggested that Ki-67 levels were significantly decreased in TIM-knockdown tissues compared to those in control tissues (Fig. 3d). Surprisingly, we found that two mice in the shNC group and one mouse in the shTIM group developed lung metastases. The 4T1 shNC syngeneic mice showed multiple pulmonary metastases in each case, whereas the 4T1 shTIM syngeneic mice showed a single pulmonary metastasis by hematoxylin–eosin staining (Fig. 3e). TIM expression was positively correlated with lung metastasis, which was also confirmed by the gene expression levels in different metastatic sites of breast cancer from the GSE46141 dataset (Additional file 1: Fig. S2). The CD8^+^ T lymphocyte infiltration level of the tumor was evaluated by flow cytometry analysis, which indicated an increased proportion of CD45^+^CD8^+^ T cells in the shTIM tumor tissues compared to that in the shNC tissues (Fig. 3f), similar to the data in the RENJI cohort mentioned previously (Fig. 2a–c). As an essential effector of antitumor immunity, cytotoxic lymphocytes require granzyme B (GB) to eliminate tumor cells through granule-mediated apoptosis [26], and GB expression levels reflect the cytotoxic activity of CD8^+^ T lymphocytes. CD8^+^ T lymphocyte infiltration and activity were both examined by immunofluorescence, and we found that TIM downregulation not only increased the percentage of CD8^+^ T lymphocytes but also increased GB release (Fig. 3g). It has been reported that antitumor immunity is positively associated with apoptosis in tumor tissue [27]. Thus, the expression levels of the apoptotic biomarker cleaved caspase 3 (CCA3) were explored. A few cells in the tumor tissues of the control group underwent apoptosis. However, strongly clustered CCA3 signals were observed in the shTIM syngeneic mice (Fig. 3h). These results indicated that TIM may facilitate breast cancer progression by attenuating CD8^+^ T lymphocyte infiltration and impairing CD8^+^ T lymphocyte activity.

To further validate the above results in vitro, we co-cultured 4T1 cells and their specific CD8^+^ T lymphocytes with or without cytotoxic antitumor activity. We performed an apoptosis assay, proliferation assay, and ELISA for IFN-γ production following co-culture (Additional file 1: Fig. S3). The cell apoptosis data obtained by flow cytometry showed that breast cancer cells gained a higher percentage of apoptosis in the TIM knockdown group than in the control group (Fig. 3i). In the proliferation assay, TIM knockdown increased the sensitivity of 4T1 breast cancer cells to the antitumor activity of CTLs (Fig. 3j). The level of IFN-γ secretion by CTLs increased in the TIM-knockdown co-culture system (Fig. 3k). These results suggest that TIM compromises the antitumor activity of CTLs.

### TIM acts as a novel regulator of PD-L1 expression and exerts oncogenic function partly through PD-L1

**Fig. 4:**
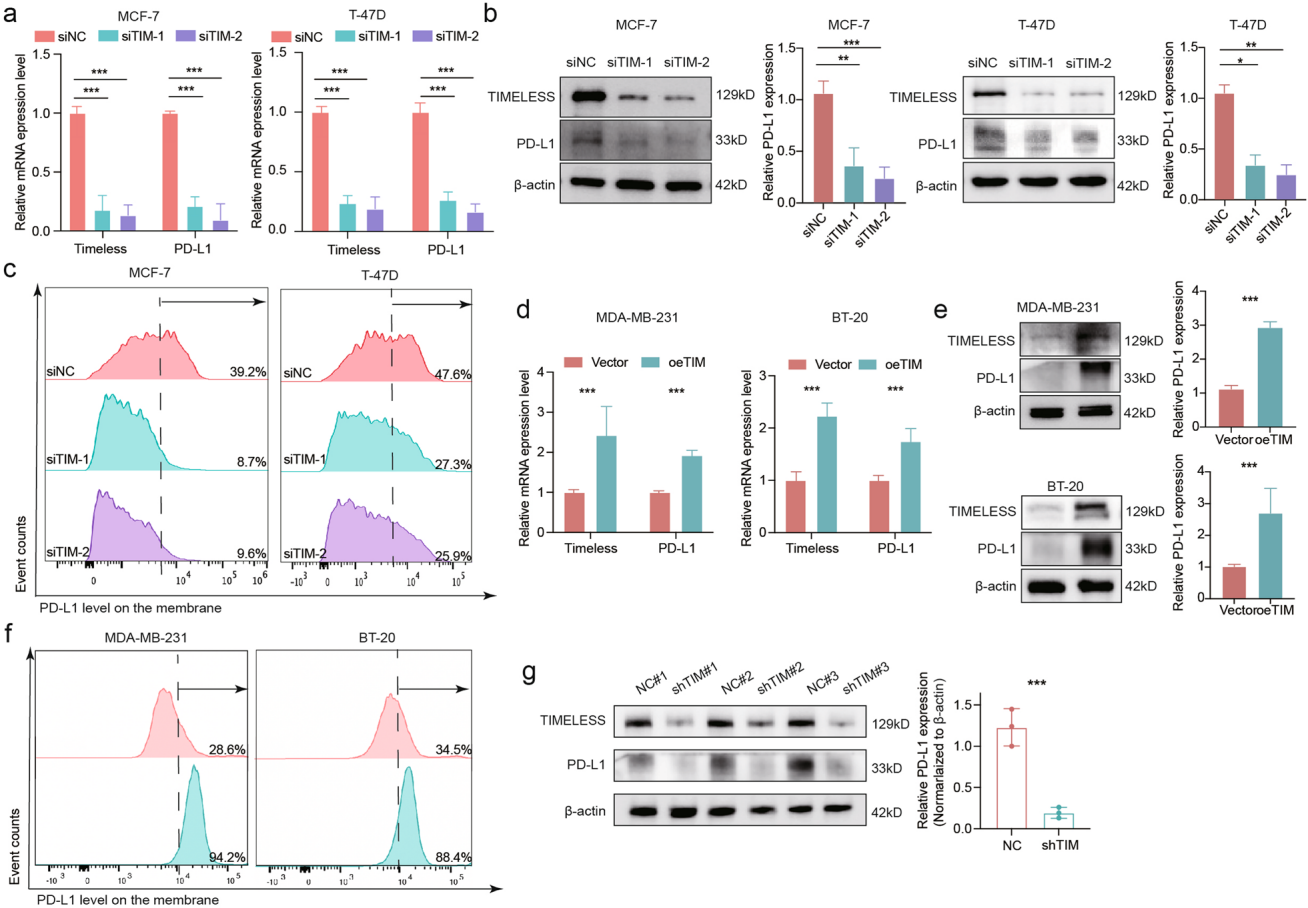
TIM regulates PD-L1 expression of breast cancer cells in vitro and in vivo. **a** The mRNA expression level of PD-L1 in breast cancer TIM knockdown cells. The mRNA expression level was analyzed by RT-qPCR. β-actin was used as an internal reference. **b** Western blot analyses of TIM and PD-L1 in breast cancer TIM knockdown cells. β-actin was used as an internal reference. **c** Representative flow cytometric plot of PD-L1 membrane expression level in breast cancer TIM knockdown cells. **d** The mRNA expression level of PD-L1 in breast cancer TIM overexpression cells. The mRNA expression level was analyzed by RT-qPCR. β-actin was used as an internal reference. **e** Western blot analyses of TIM and PD-L1 in breast cancer TIM overexpression cells. β-actin was used as an internal reference. **f** Representative flow cytometric plot of PD-L1 membrane expression level in breast cancer TIM overexpression cells. **g** Western blot analyses of TIM and PD-L1 in the syngeneic tumor mice. β-actin was used as an internal reference. Significance was tested using Mann–Whitney U-test: *p-value < 0.05; **p-value < 0.01; ***p-value < 0.001

Programmed cell death ligand 1 (PD-L1) is usually expressed on the tumor surface, is the main ligand of Programmed cell death 1 (PD-1), and can compromise T cell proliferation and active functions [28, 29]. As mentioned above, TIM can attenuate CD8^+^ T lymphocyte infiltration and immune activity, and we hypothesized that TIM may modulate PD-L1 expression. In our previous study, we found that TIM expression was higher in estrogen receptor (ER)-positive breast cancer cell lines than in ER-negative breast cancer. Therefore, we used a TIM knockdown model in ER-positive cells and a TIM-stable overexpression model in ER-negative breast cancer cell lines. RT-PCR and western blotting analyses showed that PD-L1 expression decreased after MCF-7 and T-47D cells were transfected with two different TIM siRNAs (Fig. 4a, b). A reduction in PD-L1 was also observed on the membrane by flow cytometry (Fig. 4c). Similarly, the overexpression in MDA-MB-231 and BT-20 cells enhanced PD-L1 expression at the mRNA (Fig. 4d), protein (Fig. 4e), and PD-L1 membrane levels (Fig. 4f). In the in vivo syngeneic mouse model, a decrease in PD-L1 expression due to TIM knockdown was also demonstrated at the protein level in shNC and shTIM 4T1 tumor cells (Fig. 4g).

**Fig. 5:**
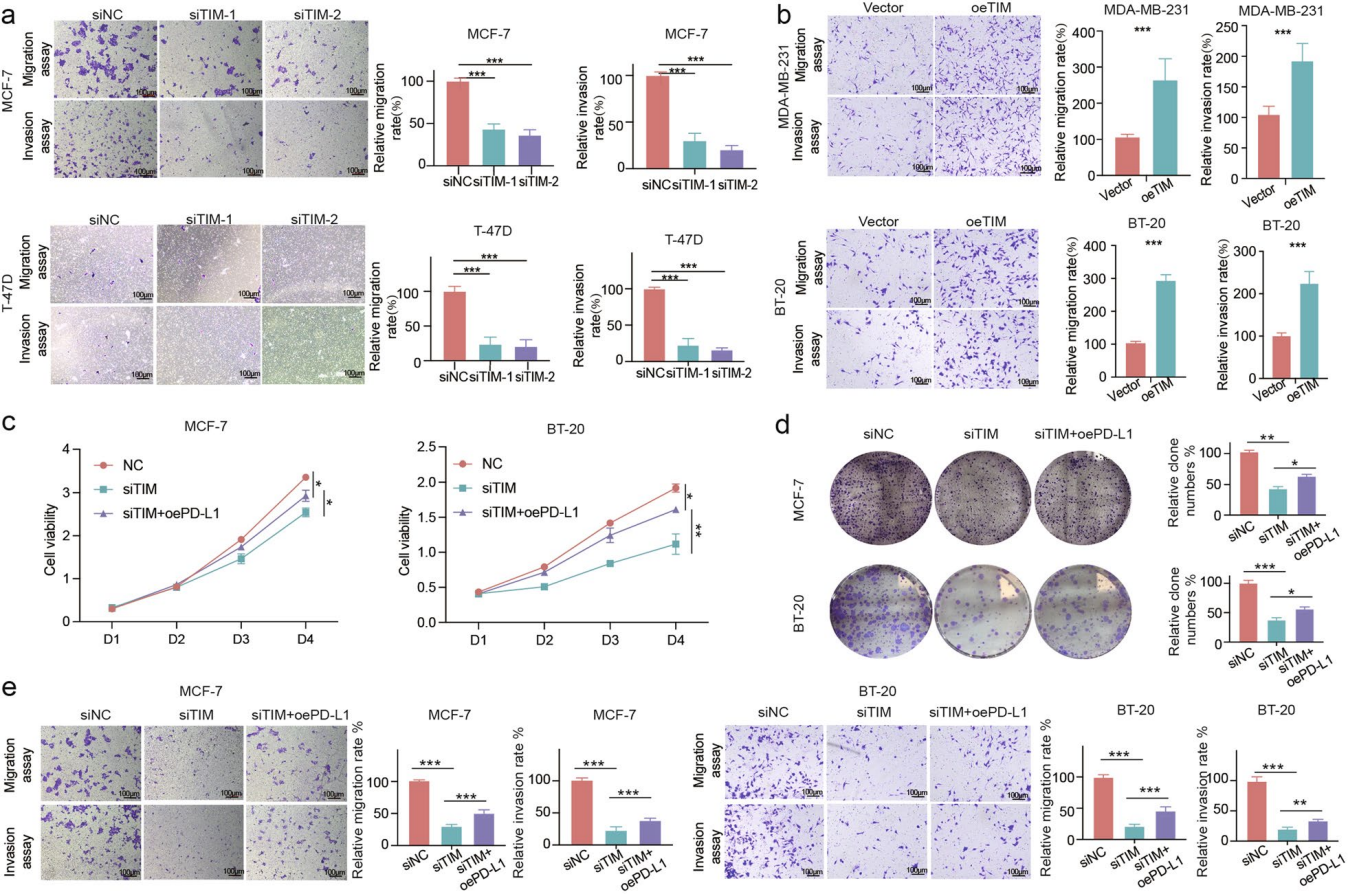
TIM affects the tumor migration and invasion ability by regulating PD-L1 expression in breast cancer cell lines. **a** The migration and invasion analyses in breast cancer TIM knockdown cells. **b** The migration and invasion analyses in breast cancer TIM overexpression cells. **c** CCK-8 assay in breast cancer TIM knockdown cells with or without PD-L1 overexpression. **d** Clonal formation assay in breast cancer TIM knockdown cells with or without PD-L1 overexpression. **e** The migration and invasion analyses in breast cancer TIM knockdown cells with or without PD-L1 overexpression. Significance was tested using Mann–Whitney U-test: *p-value < 0.05; **p-value < 0.01; ***p-value < 0.001

It has been demonstrated that TIM can promote breast cancer cell progression [30] and that PD-L1 can exert its intrinsic oncogenic function in breast cancer [31]. We postulated that TIM could affect breast cancer cell progression by influencing PD-L1 expression. Transwell migration and invasion assays confirmed that TIM knockdown reduced the migration and invasion of MCF-7 and T-47D cells (Fig. 5a). In contrast, TIM overexpression increased the migration and invasion of MDA-MB-231 and BT-20 cells (Fig. 5b). Through CCK-8 and clonal formation assays, we found that the overexpression of PD-L1 could partly rescue the decrease in proliferation ability caused by TIM knockdown (Fig. 5c, d). The transwell assay also revealed that the overexpression of PD-L1 reversed the inhibitory effects of TIM knockdown on migration and invasion ability (Fig. 5e).

### TIM regulates PD-L1 expression by interacting with c-Myc

**Fig. 6:**
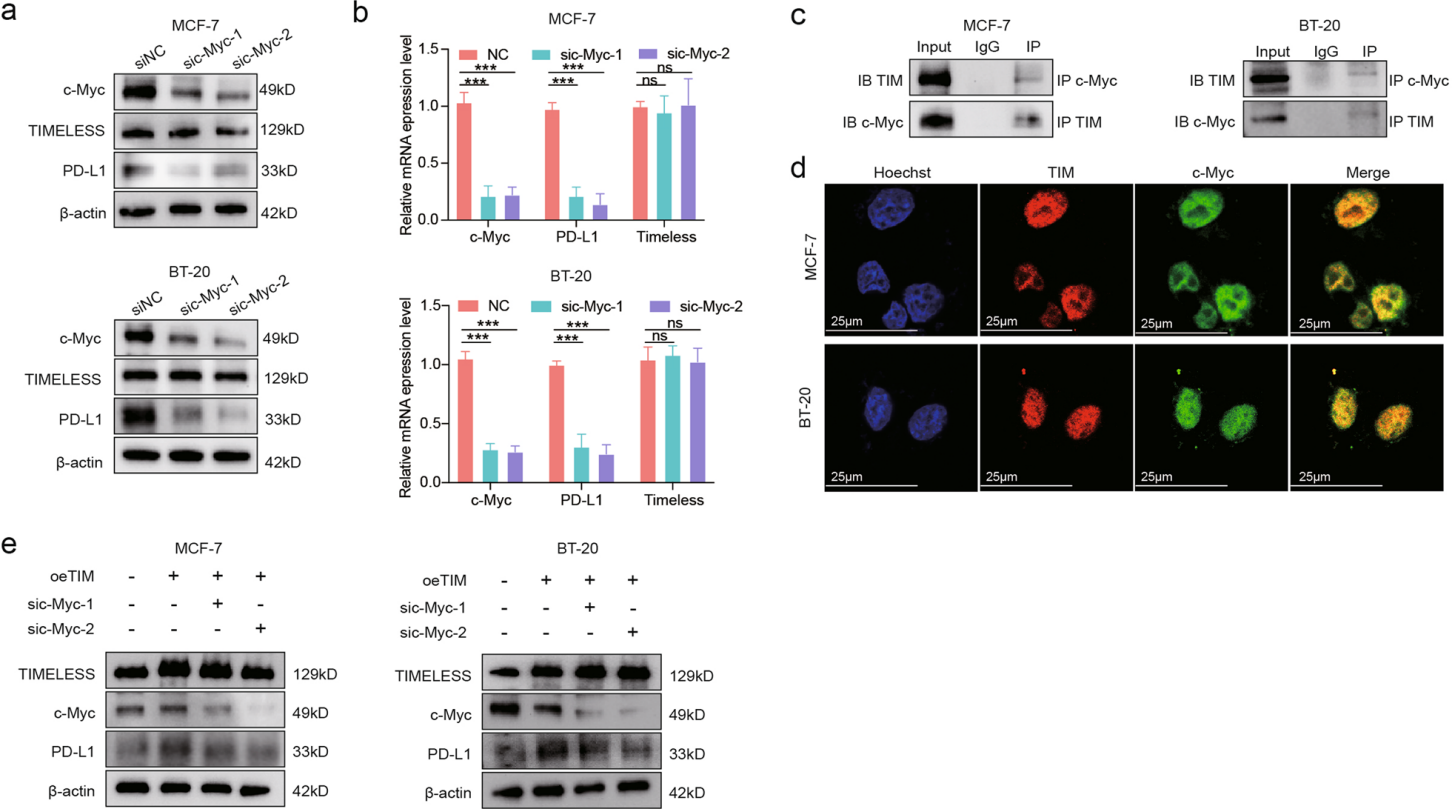
TIM enhances the transcription of PD-L1 through c-Myc. **a** The expression level of PD-L1 after knockdown of c-Myc in the MCF-7 cells and the BT-20 cells. **b** Relative mRNA levels of TIM, c-Myc, and PD-L1 after knockdown of c-Myc in the MCF-7 cells and the BT-20 cells. **c** The interaction of TIMELESS and c-Myc detected by co-immunoprecipitation in the MCF-7 cells and the BT-20 cells. **d** Immunofluorescence staining of TIM (pseudo-color: red) and c-Myc (pseudo-color: green) in MCF-7 cells and the BT-20 cells. Hoechst: nuclear counterstaining (pseudo-color: blue). The scale bar is 25 μm. **e** The expression level of PD-L1 after knockdown of c-Myc in both MCF-7 cells and BT-20 cells with TIM overexpression. Significance was tested using Mann–Whitney U-test: ns, p-value no significance; ***p-value < 0.001

Finding that TIM enhanced PD-L1 expression, we explored the mechanism by which TIM regulates PD-L1 expression. TIM interacts with several transcription factors to influence the expression of crucial oncogenic genes, thereby contributing to breast cancer progression [22]. Thus, we speculated that TIM may act as a transcriptional cofactor to regulate PD-L1 expression. According to previous studies, TIM can increase the activity of c-Myc [19], and PD-L1 can be transcribed by the recruitment of c-Myc in breast cancer [12]. We confirmed the proven results that c-Myc knockdown led to the downregulation of PD-L1 in MCF-7 and BT-20 cells (Fig. 6a). Meanwhile, the protein expression and mRNA levels of TIM were not altered by c-Myc knockdown (Fig. 6a, b). Next, we performed a co-immunoprecipitation (co-IP) assay to determine the relationship between TIM and c-Myc, which showed that TIM specifically binds to c-Myc in MCF-7 and BT-20 cells (Fig. 6c). Immunofluorescence analysis indicated that TIM co-localized with c-Myc in the nuclei of MCF-7 and BT-20 cells (Fig. 6d). Western blotting results showed that PD-L1 overexpression induced by TIM overexpression was partially inhibited by c-Myc knockdown (Fig. 6e). Taken together, our results suggest that TIM can, to some extent, enhance the transcriptional activity of c-Myc to upregulate PD-L1 expression.

### TIM serves as a novel potential biomarker for breast cancer immunotherapy, especially for anti-PD-L1 therapy

**Fig. 7:**
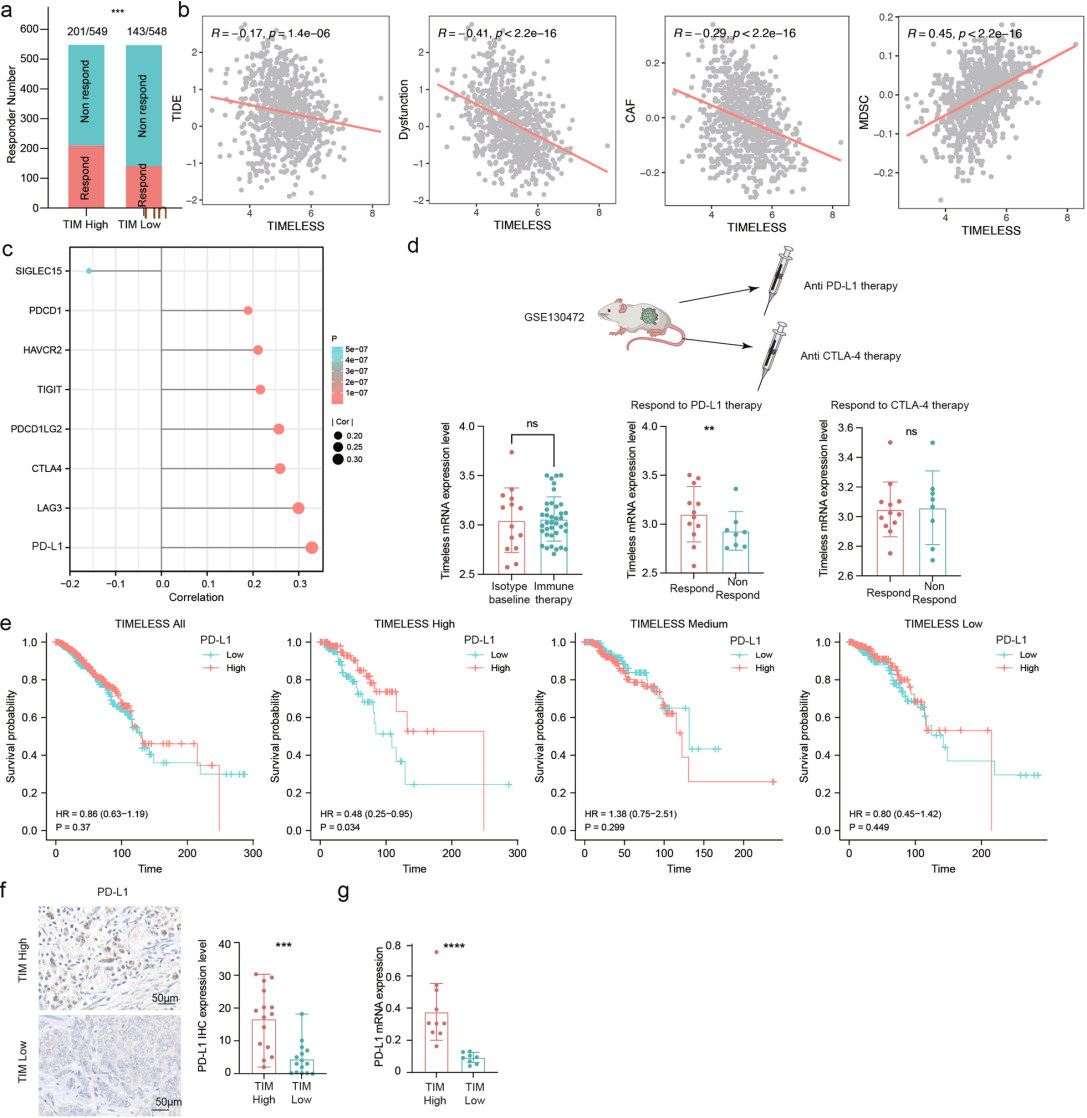
TIM could predict breast cancer immunotherapy response, especially for anti-PD-L1 therapy. **a** Rates of response to immunotherapy in TIM high and low expression groups by TIDE scores. The TIDE algorithm was used to predict the immunotherapeutic responses of BRCA patients in the TCGA dataset. Significance was tested using a χ^2^ test. **b** Correlation analyses between TIM and TIDE scores, including dysfunction of tumor infiltrating CTL, CAF and MDSC. Significance was tested using Spearman's rank correlation. **c** The lollipop chart showed the correlation between TIM and immune checkpoint genes in TCGA dataset. **d** 4T1 transplanted breast cancer mice model in GSE130472 received anti-PD-L1 therapy and anti-CTLA4 therapy (TISMO). TIM mRNA expression level didn’t change despite different immune treatments (TISMO). Better therapeutic therapy response to anti-PD-L1 therapy was seen in mice with higher TIM mRNA expression level in GSE130472 (TISMO). Significance was tested using the Student’s t-test. **e** Survival analysis of high and low PD-L1 in the total population and subgroups based on different TIM expression levels with the TCGA dataset. Significance was tested using the Cox regression analysis. **f** Representative PD-L1 immunohistochemical (IHC) images based on different TIM expression levels using tumor tissue of the RENJI cohort. The scatter plot on the right showed that PD-L1 IHC expression levels were relatively higher in patients with relatively higher TIM expression. The scale bar is 50 μm. Significance was tested using the Student’s t-test. **g** Expression of PD-L1 mRNA expression level in the RENJI cohort of patients with relatively higher TIM and lower TIM expression. The mRNA expression level was analyzed by RT-qPCR. β-actin was used as the internal reference. Significance was tested using the Student’s t-test. Significance was tested as described above: ns, p-value no significance; *p-value < 0.05; **p-value < 0.01; ***p-value < 0.001. *TIDE* tumor immune dysfunction and exclusion, *CTL* cytotoxic T lymphocyte, *CAF* tumor-associated fibroblast, *MDSC* myeloid-derived cell

Based on our findings, we hypothesized that TIM expression may be associated with the response to immune therapy in breast cancer. To further evaluate the value of TIM in predicting responsiveness to immunotherapy, we employed the Tumor Immune Dysfunction and Exclusion (TIDE) algorithm in the TCGA-BRCA dataset to calculate the immunotherapeutic response rates of patients with different TIM expression levels. Approximately 36.6% (201/549) of patients in the high TIM expression group responded to immunotherapy compared to 26.1% (143/548) in the low TIM expression group (Fig. 7a). There was a significant inverse correlation between TIDE score and TIM expression (r = − 0.17, p = 1.4e−06; Fig. 7b). Patients with high TIDE scores may benefit from immunotherapy [32]. To explore which type of immunotherapy is affected by the expression pattern of TIM, we calculated the correlation between well-known immune checkpoint genes and TIM. Most immune checkpoint genes were positively correlated with TIM, among which the most relevant was CD274, which encodes the well-known PD-L1 protein (Fig. 7c).

We further investigated the TISMO database using ICI-treated mouse models. The 4T1 transplanted breast cancer mouse model in GSE130472 was subjected to anti-PD-L1 and anti-cytotoxic T-lymphocyte-associated protein 4 (CTLA4) therapies (Fig. 7d). The TIM expression level at baseline was the same as that after immune therapy, illustrating that the TIM expression level did not change despite the different immune treatments. Upregulated TIM expression yields a better response to anti-PD-L1 therapy than to anti-CTLA4 therapy. Next, we examined the correlation between PD-L1 and TIM (Fig.S4). TIM showed the strongest correlation with PD-L1 expression in TNBC (r = 0.436, p = 1.89e−09).

To explore the relationship between TIM and PD-L1 expression in terms of prognosis, we evaluated the effect of PD-L1 on overall survival (OS) based on TIM expression levels using the TCGA dataset. The survival analysis revealed that PD-L1 expression was not significantly associated with OS in the entire study population (Fig. 7e). To integrate the TIM status into the survival analysis, we separated the patients into low (0–33%), medium (34–66%), and high (67–100%) subgroups. In the high TIM group, OS was significantly prolonged with increased PD-L1 expression. In the medium and low TIM groups, the difference was not statistically significant. The assays using tumor tissue from the RENJI cohort also showed higher levels of PD-L1 at both protein (Fig. 7f) and mRNA levels (Fig. 7g) in the high TIM expression group. These results implied that breast cancer patients with high TIM expression may be more sensitive to anti-PD-L1 therapy.

## Discussion

**Fig. 8:**
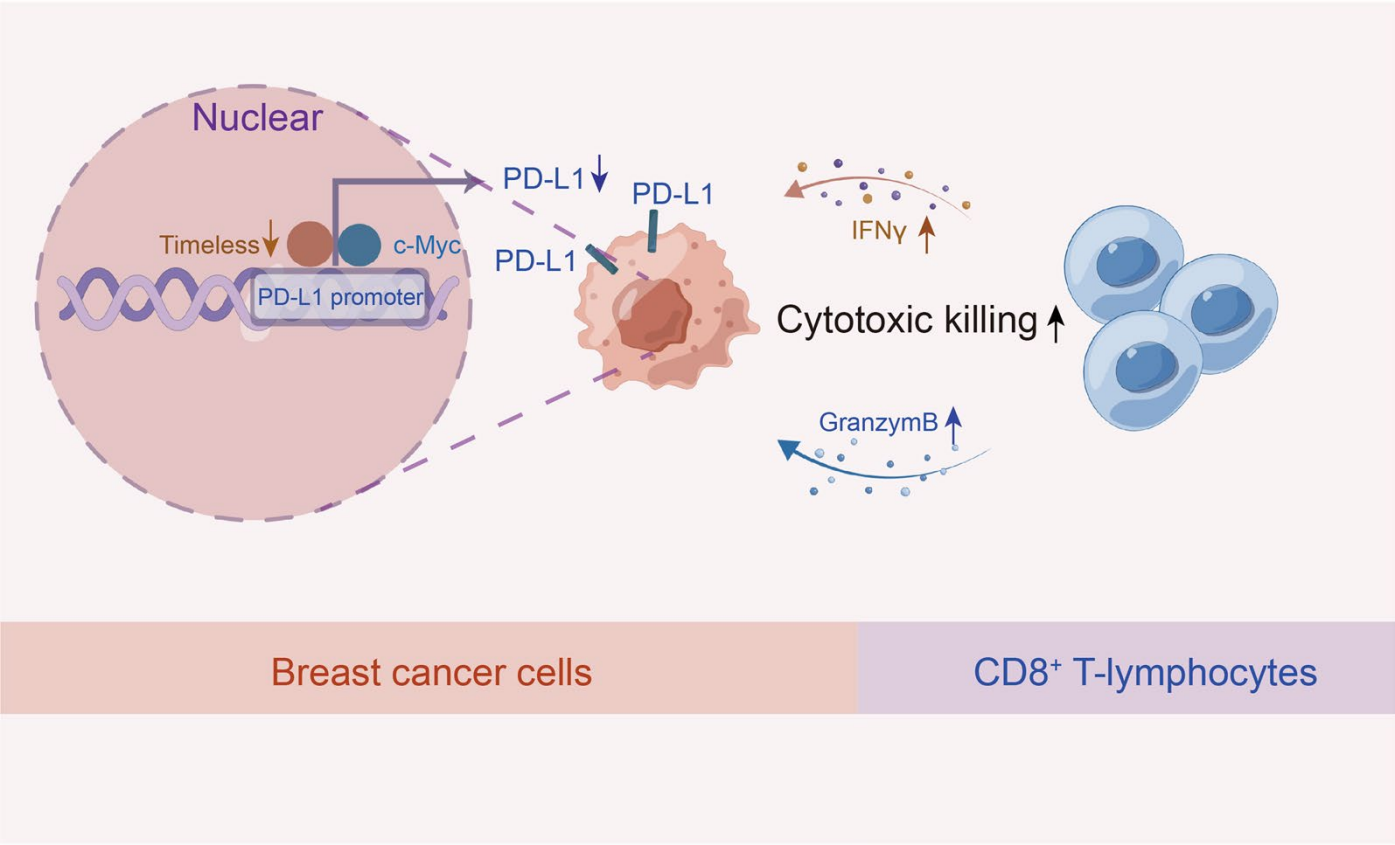
Schematic diagram of how TIM regulates tumor immunity intrinsically and extrinsically

In this study, we found that TIM participated in immune suppression by inhibiting CD8^+^ T lymphocyte infiltration and immune activity (Fig. 8). In vitro and in vivo experiments further indicated that TIM may be a novel regulatory molecule for PD-L1 transcription through c-Myc. We confirmed the intrinsic and extrinsic oncogenic roles of PD-L1 in breast cells. TIM partially facilitates breast cancer cell proliferation and invasion by upregulating PD-L1 expression. We also observed a positive association between the expression of TIM and the response to anti-PD-L1 therapy. Taken together, our findings provide new insights into the regulatory mechanisms of intrinsic PD-L1 expression and identify TIM as a promising molecular biomarker for breast cancer immunotherapy.

TIMs are traditionally regarded as circadian rhythm genes. However, it also functions as a tumorigenic gene in various cancers, including breast cancer. Our previous study showed that TIM could promote breast cancer growth partly by regulating the sphingolipid synthesis pathway [22]. Wu et al. reported that TIM could promote breast cancer cell proliferation and invasion by boosting the self-renewal of cancer stem cells [19]. Other studies have also shown that TIM could promote the growth of MCF-7 and MDA-MB-468 cells by protecting replication forks from replication stress [20, 21]. Our current study is the first to reveal that the increasing tumorigenicity of TIM and its immunosuppressive effect were partially caused by the upregulation of PD-L1 expression. We found that the apoptosis rate was higher in the siTIM group than in the control group, both in vitro and in vivo. When cytotoxic T lymphocytes were added in vitro, apoptosis rates were even higher in the siTIM group, indicating that TIM inhibited antitumor immunity. These results suggest that TIM plays a critical role in the devastating immune response in the breast cancer immune microenvironment. Collectively, our findings broaden the understanding of TIM in breast cancer progression and suggest that TIM may be a promising target for treating breast cancer.

PD-L1, a well-studied immune checkpoint molecule, plays a crucial role in promoting immune evasion of various malignant tumors by suppressing the immune activity of CD8^+^ T cells [9, 33]. Recently, researchers have focused on the intrinsic effects that promote cancer progression [31]. Our study showed that the intrinsic expression of PD-L1, independent of its interaction with PD-1 on the T-cell surface, could inhibit its antitumor properties. These findings are consistent with those of previous studies showing that intrinsic PD-L1 expression promotes cancer cell progression by orchestrating glucose metabolism, autophagy, and PI3K/AKT/mTOR signaling [34, 35].

Many studies have explored the modulatory mechanisms of PD-L1 expression, both transcriptionally and post-translationally. STAT1, STAT3, NF-κB, HIF-1α, and CD44 have been demonstrated to activate PD-L1 transcription. MARVEL Transmembrane Domain Containing (CMTM) 6 and CMTM4 have been reported to increase PD-L1 stability by reducing PD-L1 ubiquitination [36, 37]. Felsher et al. found that c-Myc interacts with the PD-L1 promoter to increase its transcription and help escape immunosurveillance [38]. One study demonstrated that TIM enhances the transcriptional activity of c-Myc in MCF-7 and T-47D cells [19]. However, little is known about the relationship between TIM and PD-L1 and the role of TIM in immune regulation. Our current study found that TIM interacts with c-Myc to improve the c-Myc-induced transcription of PD-L1. It is well acknowledged that c-Myc is a master regulator of both tumorigenesis and immune privilege [38]. Consistent with a recent study, c-Myc is required for T lymphocyte status [40]. Notably, our study established that TIM may be a new regulatory molecule for PD-L1 expression. Moreover, the modulatory axis TIM/c-Myc/PD-L1 in breast cancer has not yet been validated.

Tumor immunotherapy is becoming increasingly critical, particularly for advanced tumors. ICI targeting PD-L1 or PD-1 has provided substantial benefits to patients with advanced breast cancer. However, PD-L1-negative tumors are insensitive to immunotherapy. TIM increases PD-L1 expression and impairs the function of CD8^+^ T cells. It is well known that the immune system is highly dependent on the time-of-day [41]. Circadian clock disorders may hamper immune functions [42]. Our investigation showed that TIM, a key component of the circadian clock genes, is positively related to PD-L1 inhibitor sensitivity. Patients with high TIM levels might be potential candidates for immunotherapy, which may guide the use of immunotherapy. However, the definite network between TIM expression and circadian rhythm dysfunction requires further in-depth exploration. Taken together, our findings establish that TIM and its modulatory pathways may serve as new therapeutic targets to boost the response to PD-L1 treatment in patients with breast cancer.

## Conclusion

In summary, we revealed that the circadian gene TIM enhances PD-L1 transcription and facilitates the aggressiveness and progression of breast cancer through the intrinsic and extrinsic roles of PD-L1 overexpression. Our findings provide a novel therapeutic strategy to treat breast cancer by targeting the oncogenic effect of TIM, which may be used as a potential biomarker for anti-PD-L1 immunotherapy.

## Fundings

The conduct of this study was sponsored and funded by National Natural Science Foundation of China (No. 82173115 and 82103695), Clinical Research Plan of Shanghai Hospital Development Center (No. SHDC2020CR3003A), Science and Technology Commission of Shanghai Municipality (No. 20DZ2201600), Beijing Foundation of Medicine Award (No. YXJL-2020-0941-0737), Shanghai Municipal Key Clinical Specialty, Shanghai ‘Rising Stars of Medical Talent’ Youth Development Program for Outstanding Youth Medical Talents (No. 2018-16), Shanghai Rising-Star Program (No. 22QC1400200), Multidisciplinary Cross Research Foundation of Shanghai Jiao Tong University (No. YG2019QNA28), Clinical Research Innovation Nurturing Fund of Renji Hospital and United Imaging (No. 2021RJLY-002) and Shanghai Sailing Program (No. 22YF1424500).3

## Supplementary Information

**Additional file 1: Figure S1.** Bioinformation analysis of TIM. **Figure S2**. The expression level of TIM in different metastatic sites. **Figure S3**. The protocol and detection using mouse breast cancer cell line 4T1 co-cultured with their specific CTLs or CD8^+^ T cells in the siNC group and siTIM group. **Figure S4**. The correlation between PD-L1 and TIMELESS in different breast cancer subtypes. **Table S1**. RNA sequences used in this study. **Table S2.** Stable cell line sequences. **Table S3**. DNA Oligo Sequences used in this study.

## Abbreviations

CCK-8: Cell counting kit-8
CMTM: MARVEL transmembrane domain containing
co-IP: Co-immunoprecipitation
CTL: Cytotoxic T lymphocyte
CTLA4: Cytotoxic T-lymphocyte-associated protein 4
ER: Estrogen receptor
ERAD: Endoplasmic reticulum-associated degradation
GSEA: Gene set enrichment analysis
GSK3b: Glycogen synthase kinase 3b
ICI: Immune checkpoint inhibitors
IHC: Immunohistochemistry
KEGG: Kyoto Encyclopedia of Genes and Genomes
MHC: Major histocompatibility complex
NAL: Neoantigen load
NCBI: National Center for Biotechnology Information
OS: Overall survival
PD-1: Programmed cell death 1
PD-L1: Programmed cell death 1 ligand 1
PFA: Paraformaldehyde
RT-qPCR: Quantitative real-time PCR
shRNA: Short hairpin RNA
siRNA: Small interference RNA
Sp1: Specificity protein 1
TCGA: The cancer genome atlas
TIDE: Tumor immune dysfunction and exclusion
TIM: TIMELESS
TMB: Tumor mutant burden
TME: Tumor microenvironment
TPM: Transcripts per million
