# Supplementary material for: TIMELESS upregulates PD-L1 expression and exerts an immunosuppressive role in breast cancer
Source: J Transl Med. 2023 Jun 20;21:400. doi: 10.1186/s12967-023-04257-6 (PMC10280842; doi:10.1186/s12967-023-04257-6)
Supplement: Supplementary file 1 — Additional file 1: Figure S1. Bioinformation analysis of TIM. Figure S2. The expression level of TIM in different metastatic sites. Figure S3. The protocol and detection using mouse breast cancer cell line 4T1 co-cultured with their specific CTLs or CD8+ T cells in the siNC group and siTIM group. Figure S4. The correlation between PD-L1 and TIMELESS in different breast cancer subtypes. Table S1. RNA sequences used in this study. Table S2. Stable cell line sequences. Table S3. DNA Oligo Sequences used in this study. [file 12967_2023_4257_MOESM1_ESM.docx]

| File | Caption | Page |
| --- | --- | --- |
| Supplementary figure 1 | Bioinformation analysis of TIM | 1 |
| Supplementary figure 2 | The expression level of TIM in different metastatic sites | 2 |
| Supplementary figure 3 | The protocol and detection using mouse breast cancer cell line 4T1 co-cultured with their specific CTLs or CD8^+^ T cells in the siNC group and siTIM group | 2 |
| Supplementary figure 4 | The correlation between PD-L1 and TIMELESS in different breast cancer subtypes | 2 |
| Supplementary Table 1 | RNA sequences used in this study | 3 |
| Supplementary Table 2 | Stable cell line sequences used in this study | 3 |
| Supplementary Table 3 | DNA Oligo Sequences used in this study | 3 |

# Supplementary figure 1. Bioinformation analysis of TIM


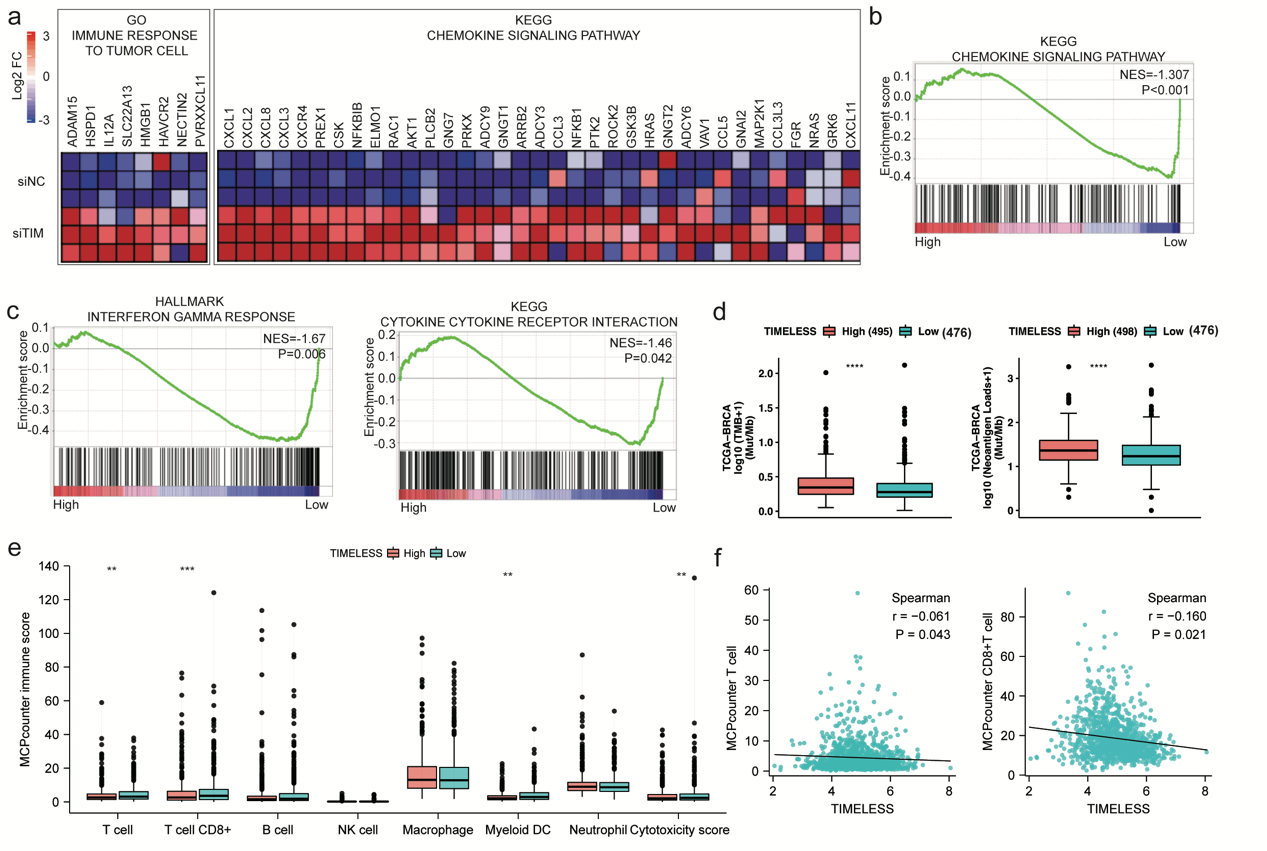


# Supplementary figure 2. The expression level of TIM in different metastatic sites


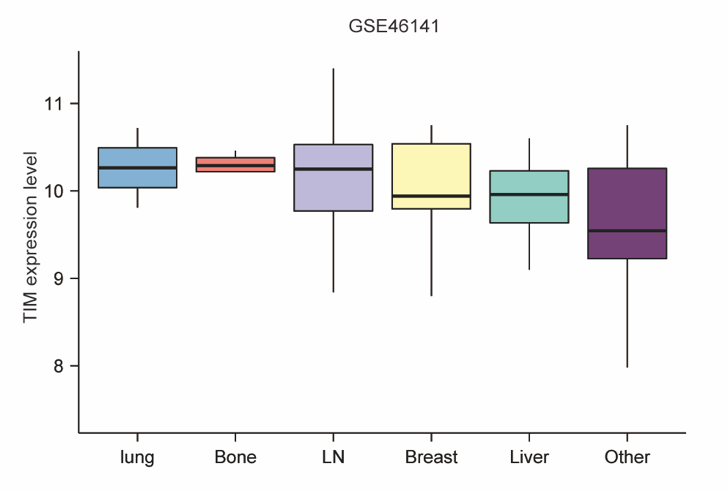


# Supplementary figure 3. The protocol and detection using mouse breast cancer cell line 4T1 co-cultured with their specific CTLs or CD8^+^ T cells in the siNC group and siTIM group.


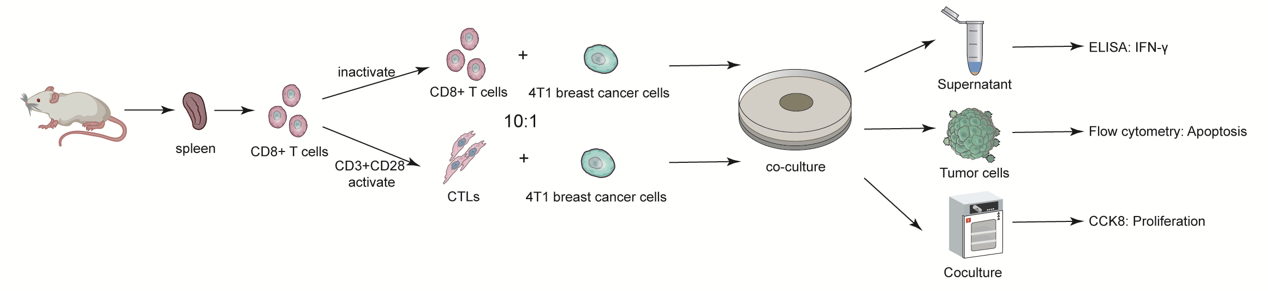


# Supplementary figure 4. The correlation between PD-L1 and TIMELESS in different breast cancer subtypes.


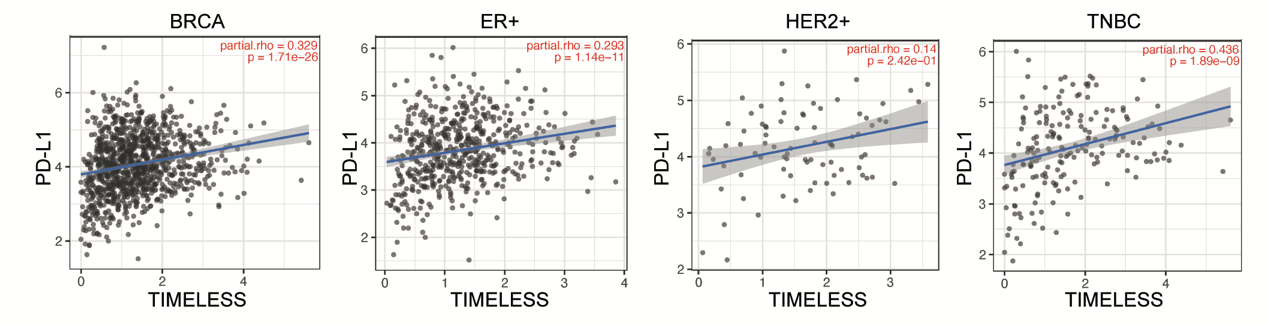


# Supplementary Table 1. RNA sequences used in this study

| Sequences for siRNA | | |
| --- | --- | --- |
| Gene | Sense (5’-3’) | Antisense (5’-3’) |
| h-siTIM-1 | CCAAAUACAUCCUGGGCAATT | UUGCCCAGGAUGUAUUUGGTT |
| h-siTIM-2 | GCUAGAGAUUGUCUCCCUUTT | AAGGGAGACAAUCUCUAGCTT |
| h-sic-Myc-1 | CCUGAGACAGAUCAGCAACAA | UUGUUGCUGAUCUGUCUCAGG |
| h-sic-Myc-1 | GGAACAAGAAGAUGAGGAA | UUCCUCAUCUUCUUGUUCC |
| m-siTIM-1 | GGAGAAGAGUAUCGAUGAUTT | AUCAUCGAUACUCUUCUCCTT |
| m-siTIM-2 | GAGCAGUCGUAUCAUCAAATT | UUUGAUGAUACGACUGCUCTT |

h refers to human and m refers to mouse.

# Supplementary Table 2. Stable cell line sequences used in this study

| Sequences for shRNA and plasmids | | |
| --- | --- | --- |
| Gene | Sense (5’-3’) | Antisense (5’-3’) |
| h-oeTIM | CCAAAUACAUCCUGGGCAATT | UUGCCCAGGAUGUAUUUGGTT |
| m-shTIM | GCUAGAGAUUGUCUCCCUUTT | AAGGGAGACAAUCUCUAGCTT |

h refers to human and m refers to mouse.

# Supplementary Table 3. DNA Oligo Sequences used in this study

| Primers for qPCR | | |
| --- | --- | --- |
| Gene | Forward primer | Reverse primer |
| Timeless | AGAGGAGGAGGAGGAGGAAGAGG | CAGCCGCACAATCGCCACTC |
| CD8a | ATGGCCTTACCAGTGACCG | AGGTTCCAGGTCCGATCCAG |
| PD-L1 | GGCATTTGCTGAACGCATTT | TGCAGCCAGGTCTAATTGTTTT |
| c-Myc | GGCTCCTGGCAAAAGGTCA | CTGCGTAGTTGTGCTGATGT |
| Actin | CATGTACGTTGCTATCCAGGC | CTCCTTAATGTCACGCACGAT |
